# User Real-Time Influence Ranking Algorithm of Social Networks Considering Interactivity and Topicality

**DOI:** 10.3390/e25060926

**Published:** 2023-06-12

**Authors:** Zhaohui Li, Wenjia Piao, Zhengyi Sun, Lin Wang, Xiaoqian Wang, Wenli Li

**Affiliations:** 1 School of Maritime Economics and Management, Dalian Maritime University, Dalian 116026, China; 2 Graduate School of Information, Waseda University, Kitakyushu 808-0135, Japan; 3 Zhejiang Provincial Military Command, Hangzhou 310002, China; 4 School of Economics and Management, Dalian University of Technology, Dalian 116024, China

**Keywords:** social network analysis, network structure, interaction behavior, user influence

## Abstract

At present, the existing influence evaluation algorithms often ignore network structure attributes, user interests and the time-varying propagation characteristics of influence. To address these issues, this work comprehensively discusses users’ own influence, weighted indicators, users’ interaction influence and the similarity between user interests and topics, thus proposing a dynamic user influence ranking algorithm called UWUSRank. First, we determine the user’s own basic influence based on their activity, authentication information and blog response. This improves the problem of poor objectivity of initial value on user influence evaluation when using PageRank to calculate user influence. Next, this paper mines users’ interaction influence by introducing the propagation network properties of Weibo (a Twitter-like service in China) information and scientifically quantifies the contribution value of followers’ influence to the users they follow according to different interaction influences, thereby solving the drawback of equal value transfer of followers’ influence. Additionally, we analyze the relevance of users’ personalized interest preferences and topic content and realize real-time monitoring of users’ influence at various time periods during the process of public opinion dissemination. Finally, we conduct experiments by extracting real Weibo topic data to verify the effectiveness of introducing each attribute of users’ own influence, interaction timeliness and interest similarity. Compared to TwitterRank, PageRank and FansRank, the results show that the UWUSRank algorithm improves the rationality of user ranking by 9.3%, 14.2%, and 16.7%, respectively, which proves the practicality of the UWUSRank algorithm. This approach can serve as a guide for research on user mining, information transmission methods, and public opinion tracking in social network-related areas.

## 1. Introduction

The social network market has rapidly developed thanks to the digital economy era and the rapid growth of Mobile Internet use. It has become a significant channel and carrier for maintaining social relationships and distributing news in human society. Weibo, one of the most popular social networking platforms today, has emerged as a significant means for users to exchange and obtain information. Its openness, immediacy of contact, simplicity of content, and strong interaction make it an attractive platform for government operations [1], and its effect on leading public opinion in public events is obvious. Weibo has also ushered in a new era of information transmission and is welcomed by internet users. As the source of news dissemination, the importance of the user who publishes and spreads the information is self-evident. To a certain extent, it determines the dissemination path and scope of the information. The information and comments posted by certain user groups in response to public opinion events are soon known by their followers [2], prompting a significant number of users to discuss and pay attention to them. These user groups include those with high attention and discourse power. Through social behaviors like forwarding, each information recipient turns into a new information publisher once more, and this fission-spreading process causes the original users’ comments to have a significant social impact quickly and to influence public opinion. In general, a few users called “influencers” can create a chain reaction of influence that is based on a word-of-mouth approach and reach a very large scale of users [3]. More influential users have a greater reach and impact on society when they post information compared to less influential users. In this work, our definition of influence is based on the following intuition: if a user is both followed and interacted with by many users, then we consider this user to be influential. Previous studies have shown that the data generated in the modern world is enormous and growing exponentially [4]. The 51st Statistical Report on the Development of the Internet in China shows that as of December 2022, the number of netizens in China has reached 1.067 billion. In this digital age, information in various fields of our lives is increasing significantly. Taking the number of Weibo users as an example, according to the Sina Weibo User Development Report, as of May 2023, the monthly active users on Weibo have reached 586 million, a year-on-year increase of 13 million, and the daily active users have reached 252 million, a year-on-year increase of 3 million [1]. It can be seen that the scale of Weibo users is huge, and the number of users is continuously increasing. Therefore, in this era of data explosion, the main challenge faced by various organizations, industries, and other business departments is how to design appropriate technologies to handle large amounts of data to ensure effective and efficient decision-making. However, facing such a large amount of user data, existing influence evaluation algorithms cannot objectively and accurately calculate the influence of users. Thus, how to effectively evaluate the influence of users in social networks has become a problem that needs to be addressed.

Given that social networks are important channels for social relationship maintenance and information dissemination and can have far-reaching effects on both national security and social development, many scholars at home and abroad have researched the influence of social network users and proposed evaluation algorithms on user influence. For example, the HIUD algorithm [5], which measures users’ own attributes using the number of followers and blogs; the TwitterRank algorithm [6], which considers the type of topic content and network structure; the FansRank algorithm [7], which focuses on the behavior of followers during information dissemination, and some PageRank-like algorithms [8], which consider the topology of social networks. Although the above algorithms have their own research focus, they all suffer from a lack of comprehensive consideration. They simply use one or two indicators to measure the influence of users, ignore the interest of users in specific topics, and do not take into account the time-varying propagation characteristics of influence, which may lead to unrealistic and inaccurate evaluation results. Therefore, this paper improves the PageRank algorithm by combining the basic influence of users themselves and the dynamic interaction mechanism of users’ information dissemination process and introduces the similarity between users’ interests and topics to propose a dynamic measurement model of user influence based on interest preferences. Compared with other algorithms, the UWUSRank algorithm is more comprehensive in considering factors and more realistic and objective in its evaluation.

Analyzing the influence of users on Weibo topics is important for the study of social networks. Firstly, influential users are the driving force behind the continuous spread of topics and their expansion [9]. By studying the influence of Weibo users, the spreading pattern of Weibo topic information can be revealed. Secondly, Weibo topics have complex social attributes during dissemination [10]. Studying the influence of Weibo users helps to understand the social properties of social networks. Finally, the influence of Weibo users varies, and users need to be recommended as high-quality and influential users in order to get valuable and interesting information [11]. Identifying influential users helps to recommend high-quality friends with common interests on Weibo. Therefore, this study is highly significant for topic information dissemination, Weibo friend recommendation, and online opinion monitoring.

Our main contributions are as follows:

1. The UWUSRank algorithm proposed in this article provides a more systematic and comprehensive analysis of users’ own attribute factors and their interactive behavior. By improving the PageRank algorithm, we dynamically describe the user’s influence from two aspects: the user’s own influence and their interaction influence;
2. When analyzing the structure of social networks, this paper innovatively incorporates two indicators: connection tightness and interaction timelines. The tightness of connections reflects the varying degrees of correlation between users; The interaction timelines can be used to calculate the contribution of interaction behavior at different times to blogs. By considering the degree of user association and time factors, different interaction contribution rates of users can be calculated, making the interaction influence value of users more accurate;
3. We introduce the correlation between user interests and topic content in the context of the topic. By considering the significant differences in the diffusion of different types of topic information among users, our model can evaluate the actual influence of Weibo users more reasonably.

## 2. Related Works

The incoming and outgoing links of online pages and the user interaction in social networks are comparable; hence, the link analysis principle has also been used to assess the influence of user nodes on Weibo. The PageRank algorithm, which effectively ranks the value of web pages, i.e., the importance of each node in the network, is currently the source of many important link relationship analyses. Existing research typically combines the link analysis method with social network characteristics to suggest better approaches for evaluating the influence of social network users. The algorithm is improved primarily from user node characteristics, user interaction patterns, and user interests.

Influence studies based on user node characteristics typically employ metrics such as the number of followers, blogs, retweets, and mentions as indicators. For instance, Zhang et al. [12] proposed a multi-angle user importance calculation method with event-specificity. The importance of users is measured by considering four levels: user level, fan level, Weibo level and event level. Zhao et al. [5] proposed an influential user discovery algorithm based on user behavior analysis. They designed a user influence calculation formula, which combined several indicators, including the number of fans, user activity and fan activity. When Alp et al. [13] examined the number of blog retweets with the number of followers for individual users, they discovered that there was no correlation between the two. Based on the above findings, it is discovered that while the number of users’ followers is thought to be a crucial variable in determining user influence, it does not accurately represent user influence. The aforementioned influence calculation techniques ignore the external context and solely consider the user’s personal factors, resulting in excessive bias in the evaluation findings.

The method based on user interaction behavior mainly estimates node influence based on graphical features such as node degree and position in social networks. Community Scale-Sensitive Maxdegree (CSSM) [14] aims to determine the influential nodes in a network. Despite the appealing results of CSSM, some countercases can take place easily because of spammers and fake friends. In order to tackle this problem, a new model Kshell and the Community centrality model(KSC) [15], is proposed. It takes into consideration both the internal and external properties of the node. In spite of the proven fact that the Kshell property has the best influence indication, it is designed for unweighted graphs, which means all the graph edges are treated equally. Lin et al. [16] introduced user interaction attributes in user relationship networks and proposed a user influence ranking algorithm based on interaction behaviors (IB-UIR) to measure users’ influence on hot issues. Based on PageRank, the IB-UIR algorithm comprehensively considers users’ behaviors and the time interval between behaviors and calculates user influence iteratively in the user interaction network. Li et al. [17] constructed a repetitive forwarding model to characterize the diffusion process in social networks and theoretically analyzed the influence of users below and above the diffusion threshold, providing ideas for modeling and estimating the influence of social network users. Bo et al. [18] proposed a PageRank-like method that utilizes the closeness method to model transition probabilities that capture influence propagation on a social network. This method generally performs well but faces challenges in the lower follower percentiles of the social network. M et al. [19] detected influential users by network topology obtained from communication relationships among users, link analysis approach, and user profile features. Their experimental result showed that the trending topic influencers could be detected using the interaction relationships and user’s features. Although the PageRank algorithm that combines the user’s own attributes and interaction behavior attributes can measure user influence more accurately, it tends to ignore the community structure characteristics of behavior construction, leading to inaccurate measurement of the strength of the influence that various node interactions have on user influence.

According to the feature that Weibo information dissemination has topic differentiation, users can be divided into different communities before influence calculation. Abd Al-Azim et al. [20] divided users into groups based on the similarity of their interests and constructed a propagation network graph for each interest group, and they ranked the influence in each group by considering the nodes with higher influence to be located at the center of the interest group. Gomasta et al. [21] considered the impacts of topical similarities from direct and indirect neighbors of the users, which produced better results. Eliacik et al. [22] extended the original PageRank algorithm with three metrics: User Trust, Influence Period and Interest Topic Similarity, and their method was shown to be more effective in finding influential users in the microblogging community. Zhang et al. [23] proposed the VoteRank algorithm to identify spreaders in a network based on a voting mechanism. Kumar et al. [24] further extended VoteRank and designed the NCVoteRank algorithm, which takes the coreness value of neighbors into account for the voting. Lee et al. [25] proposed the MPHAT model, an extension of HAT, to jointly model the topic-specific hub users, authority users, their topical interests and platform preferences. These methods concentrate on user interaction, but they disregard the relevance between user interests and engagement in topics, making it difficult to identify users who consistently have a significant impact on a certain topic domain.

In conclusion, there are several issues that need to be addressed: (1) the inner rules of individual behavior are not fully revealed when starting from target user attributes; (2) the analysis based on social network structure fails to consider the connection density and interaction time difference of nodes; (3) the relevance of users’ interest preferences to the content of Weibo topics has not been explored; (4) the contribution of user interaction to user influence changes over time, which is often ignored. Therefore, this paper comprehensively considers the influence of Weibo users based on their own characteristic attributes and interaction behaviors. Meanwhile, we introduce user interest values in the context of topics. Finally, we propose a UWUSRank algorithm based on the user’s own influence and interaction influence while considering user interest. The user’s own influence replaces the initial influence distributed equally in the PageRank algorithm, solving the situation that each node has the same initial PR value in the PageRank algorithm and excluding the interference of fake followers on influence. The evaluation of interaction behavior can solve the problem of equal distribution of the PR value of the web page’s outgoing links in the PageRank algorithm, and users with strong interaction relationships will get a higher proportion of influence value. Additionally, we introduce the similarity between users’ interests and topics to measure the influence value of users more comprehensively and accurately in the process of topic dissemination.

## 3. Indicator System Establishment and UWUSRank Model Construction

The majority of the hot events of recent social concern are transmitted in the form of topics on Weibo, one of the most popular social network media today [26]. Users engage in the discussion of the topic by posting blogs, which have an impact on other users’ perceptions of the events and lead to online interactive behaviors, a phenomenon that reflects the influence of Weibo users [27]. The higher the influence of the users within a Weibo topic, the more people it affects and the greater the impetus to information dissemination. In the user interaction network, the more the number of other user nodes involved, the wider the population covered by the information is represented [28]. Hence, the user’s influence should be quantitatively defined from two perspectives: the user’s own influence and the user’s interaction behavior influence.

**Figure 1:**
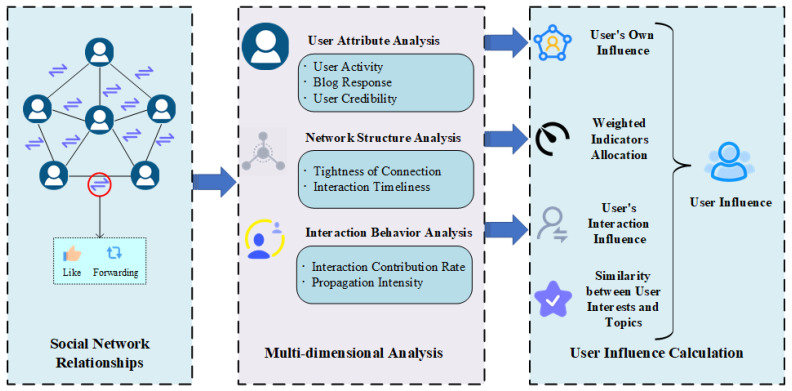
Weibo user influence calculation framework.

This paper collects and monitors trending topics from Weibo and examines the size of the communication network, the rate of information flow, and the strength of the social connections among users who are actively involved in the topic dissemination. Then we calculate the corresponding user influence. The Weibo user influence calculation approach proposed in this paper is shown in Figure 1. The model consists of four parts: user’s own influence calculation, weighted indicators calculation, user’s interaction influence calculation, and similarity calculation between user interests and topics.

- The user’s own influence value, as the initial influence value in the improved PageRank algorithm, is composed of user activity, blog response, and user credibility. The definitions of these three indicators will be detailed in Section 3.1;
- The two weight indicators of connection tightness and interaction timeliness take into account the closeness of user relationships and the time factor of interaction behavior. They are used to calculate the interaction contribution rate of user influence for subsequent calculation of user interaction influence values. The weighted indicators will be defined in detail in Section 3.2;
- For the calculation of users’ interaction influence, this paper considers forwarding behavior and adds interaction timeliness to dynamically obtain the value of the interaction contribution rate. This part is detailed in Section 3.3;
- Section 3.4 introduces the similarity between user interests and topics. We use cosine similarity to calculate the similarity between the user’s blog content and topic content as the final weight of user influence in the context of the topic.

### 3.1. Definition of the Influence Factors of User’s Own Influence

Weibo users tend to intuitively judge the influence of other users based on some of their attributes [1]. So, it is possible to evaluate the user’s own influence with reference to their own attributes. In order to better calculate a user’s influence, three indicators are defined: user activity, blog response and user credibility, respectively, as defined below.

- User activity

User activity describes how actively a user has promoted the topic in the most recent time frame, which can be measured by the number of blogs a user has published, forwarded or liked over time. The more influential users frequently participate more actively in hot events and contribute more to their dissemination. In this study, user activity is measured by the number of recent blogs and the number of likes and retweets. The calculation formula is presented in Formula (1).

(1) $$act\left(u\right)=\frac{N_{count\left(u\right)}+N_{repost\left(u\right)}+N_{like\left(u\right)}}{T}$$

where act(u) is the activity level of user 𝑢 and T is the time period, $N_{count\left(u\right)}$ is the total number of blogs posted by user u in time period T, $N_{repost\left(u\right)}$ is the total number of reposted blogs by user u in time period T, and $N_{like\left(u\right)}$ is the total number of liked blogs by user u in time period T;

- Response to blogs

The response to a blog represents the degree to which a user’s recent blog has triggered discussion among other users. In this paper, we measure this index by analyzing the number of recent comments, retweets and likes, which is shown in Formula (2).

(2) $$res\left(u\right)=\frac{\sum_{i=1}^{N}R_{i\left(u\right)}}{T_{R\left(t\right)}}+\frac{\sum_{i=1}^{N}C_{i\left(u\right)}}{T_{C\left(t\right)}}+\frac{\sum_{i=1}^{N}L_{i\left(u\right)}}{T_{L\left(t\right)}}$$

where res(u) is the recent blog response of user u, $R_{i\left(u\right)}$, $C_{i\left(u\right)}$, and $L_{i\left(u\right)}$ are the number of retweets, comments and likes of user u’s ith blog, respectively. N is the total number of tweets posted by user u in the statistical period, $T_{R\left(t\right)}$, $T_{C\left(t\right)}$, and $T_{L\left(t\right)}$ are the total number of retweets, comments and likes of users involved in topic discussions in the statistical period, respectively;

- User credibility

The credibility of the user can also reflect the influence of the user to a great extent [29]. For example, if a user is officially certified on Weibo, his statements are viewed by more users. And people tend to trust the blogs posted by these users, making them more influential. Therefore, Weibo authentication can greatly enhance a user’s credibility in the network, making them more likely to be noticed and recognized by others. Weibo provides several authentication mechanisms, including interest authentication, identity authentication, self-publishing authentication and official authentication. This paper refers to Sun’s definition of Weibo user trustworthiness for defining the credibility of users across different Weibo authentication methods [30]. The formula for calculating user credibility aut(u) is shown in Formula (3).

(3) $$aut\left(u\right)=\{\begin{array}{c} 1.5,\;\;\mathrm{Official}\;\mathrm{Certification}\\ 1,\;\;\mathrm{Identity}\;\mathrm{Verification}\\ 0.5,\;\;\mathrm{Interest}\;\mathrm{Certification}\;\mathrm{or}\;\mathrm{Self}\;-\;\mathrm{Media}\;\mathrm{Certification}\\ 0,\;\;\mathrm{No}\;\mathrm{Certification} \end{array}$$

Then, we analyze the Weibo user’s own features and define the influence score $I_{0}\left(u\right)$ based on the user’s own attributes after normalizing the extracted user features. The calculated expression is shown in Formula (4).

(4) $$I_{0}\left(u\right)=\phi_{1}\cdot act\left(u\right)+\phi_{2}\cdot res\left(u\right)+\phi_{3}\cdot aut\left(u\right)$$

where: $\phi_{1}$ is the weight of user activity, $\phi_{2}$ is the weight of user response to blogs, and $\phi_{3}$ is the weight of user credibility;

- Optimal weight acquisition

**Figure 2:**
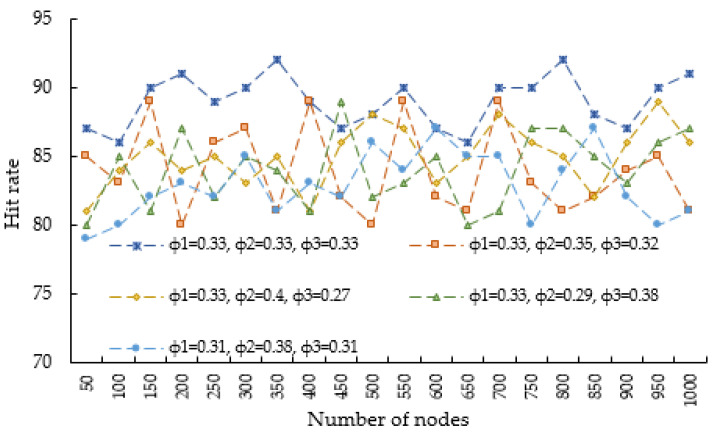
Top 5 curves with relatively good hit rate trends.

Based on past experimental experience and social network reality, different values are assigned to the weights in $I_{0}\left(u\right)$, and the assigned weights form a weight group. Multiple experiments are conducted using different weight groups so that each weight group corresponds to a user influence ranking result, and the optimal weight value is determined based on the hit rate. The calculation process of the hit rate is as follows: first, calculate the sorting result of the dataset through the influence algorithm, and then record the proportion of the first n nodes in the sorting result in the first n nodes of the benchmark data, which is the hit rate of the algorithm. Figure 2 depicts the top five curves with relatively good hit rate trends for different weight groups. Based on the experimental results, we set $\phi_{1}$ = $\phi_{2}$ = $\phi_{3}$ = 1/3, which means that the user’s activity, blog response and credibility are equally important to measure the user’s own influence.

### 3.2. Determination of Weighted Indicators

Influence propagation by users is not equally distributed to surrounding nodes, and interactions with highly connected edge weights result in greater value or strength of influence transfer between users. The connection edge weight reflects the importance of the interaction to the user’s influence. By quantitatively evaluating the tightness of the connections between network nodes and the timeliness of information exchange, we can determine the path weights in the interaction network.

1. Tightness of connection

**Figure 3:**
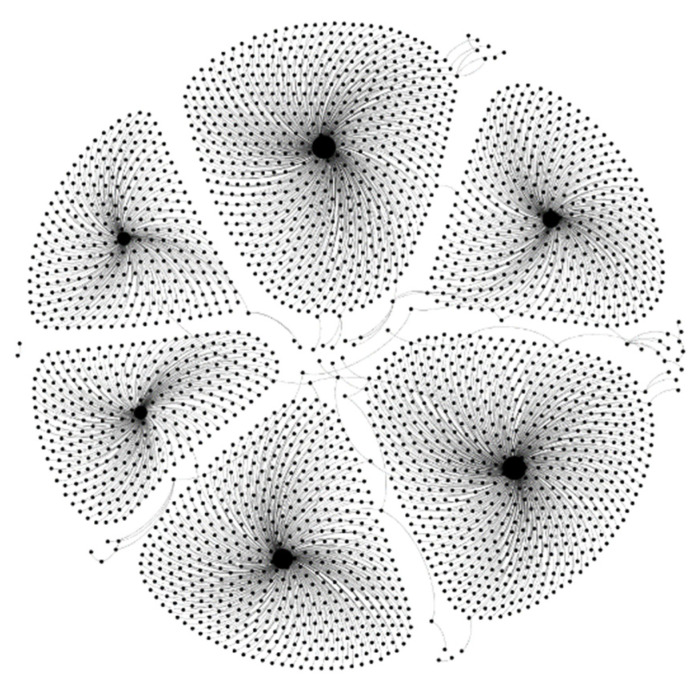
Weibo users’ community structure.

Weibo contains several intricate, overlapping community structures and covers a wide range of topics. Intra-community linkages are relatively strong, with frequent information exchanges and higher connection densities [31]. However, the connections between communities are relatively loose, which reflects the connection characteristics of users to each other, such as preference, clustering, and trust, as shown in Figure 3. Each node in Figure 3 represents a user, and the connecting lines between users represent the interaction behavior between the two users. In Weibo, there are common neighbor nodes. Based on user connection traits, two nodes in the same community typically have more identical neighbor nodes than two nodes in separate communities.

Therefore, as the number of common neighbor nodes among users increases, the possibility that users are from the same community also increases. The more frequently users interact, the closer their relationships are, and the more power users have in core positions. In social networks, the importance of a node can be measured by the influence of users who are directly adjacent to that node. However, there are differences in the influence generated by in-degree neighbors and out-degree neighbors. In-degree neighbors are users who interact with this user, and out-degree neighbors are users who this user interact with. In order to better reflect the influence of topology and interaction between nodes in social networks on users, this paper refines the influence of nodes’ in-degree neighbors and out-degree neighbors on influence propagation according to the method proposed by Fu et al. [32]. And we introduce the number of connected edges of common neighbor nodes to obtain the connection tightness between nodes. The calculation formula is provided in Formula (5).

(5) $$a_{1}\left(u,v\right)=\frac{\left|\alpha \left(u\right)\cap \alpha \left(v\right)\right|+\left|\beta \left(u\right)\cap \beta \left(v\right)\right|-\left|\alpha \left(u\right)\cap \alpha \left(v\right)\cap \beta \left(u\right)\cap \beta \left(v\right)\right|}{\left|\alpha \left(u\right)\cup \alpha \left(v\right)\cup \beta \left(u\right)\cup \beta \left(v\right)\right|}$$

where $a_{1}\left(u,v\right)$ is the proportion of the number of common neighbors among users. We distinguish between the out-degree α(i), the number of interactions produced by user i, and the in-degree β(i), which sums the number of interactions that user i received. The formula for connection tightness is shown in Formula (6).

(6) $$\gamma \left(u,v\right)=a_{1}\left(u,v\right)\times a_{2}\left(u,v\right)$$

where γ(u,v) is the connection tightness between users and $a_{2}\left(u,v\right)$ is the number of connected edges generated by the common neighbor nodes. The larger the value of γ(u,v), the closer the relationship between user u and user v, and the greater the influence transmitted when the two are interacting;

2. Interaction Timelines

User u may post multiple blogs related to the topic, and another user v may interact with user u. Therefore, using $p_{i}\left(u,v\right)$ to represent the interaction between user v and user u’s ith blog. The value of $p_{i}\left(u,v\right)$ is shown in Formula (7).

(7) $$p_{i}\left(u,v\right)=\{\begin{array}{c} 1,If\;user\;v\;interacts\;with\;the\;ith\;blog\;of\;user\;u\\ 0,If\;user\;v\;does\;not\;interact\;with\;the\;ith\;blog\;of\;user\;u \end{array}$$

Users’ focus on online public opinion can shift with time [33]. At the same time, users are more likely to be found by other users if they participate in public event conversations promptly and if posted comments are circulated promptly [34]. This suggests that earlier interactive behavior can benefit the widespread dissemination of blog information and increase the contribution value to the original user’s blog popularity. Weibo topic heat will generally decrease in the process of advancing in a short period of time [34], so a process of progressive value depreciation over time is required. Therefore, this paper employs an exponential decay function to model the influence weight of user interaction behavior on the propagation effect of blogs, which is also in line with Gotez et al.’s conclusion that the decay of blog influence follows a power-law distribution based on microblog analysis [35]. The calculation formula is presented in Formula (8).

(8) $$\lambda \left(u,v\right)=\left(\frac{\sum_{i\in S\left(u\right)}\exp(-\frac{{\hat{t}}_{i}-t_{i}{}^{0}}{\alpha })\cdot p_{i}\left(u,v\right)}{\sum p_{i}\left(u,v\right)}\right)$$

where λ(u,v) is the interaction timeliness between user u and follower user v, S(u) is the set of blogs that are posted by user u, $p_{i}\left(u,v\right)$ is the interaction relationship between user v and user u’s blog i. α is the exponential decay constant, based on the results of Gotez et al. setting α = 11 h [35]. $t_{i}{}^{0}$ is the publish time of user u’s ith blog, and ${\hat{t}}_{i}$ is the interaction time of user v’s participation in user u’s ith blog. The smaller the time interval between interactions, the greater the impact on the propagation effect of user’s blogs.

### 3.3. Definition of Influence Based on Interaction Behavior

On the Weibo social network, users can retweet, comment, and like blogs on various topics. Among these behaviors, only retweeting can achieve the function of direct information dissemination [9]. The interactive influence of users is mainly spread through the retweeting behavior of blogs [1]. Therefore, in this paper, the number of times a blog has been retweeted is used as a measure of the popularity of the blog and its author. In addition, the formal characteristics of information dissemination patterns in social networks determine that the surrounding nodes of user u differ at different times. To address the problem that previous studies do not consider the time factor when assessing user interaction influence, this paper adds interaction timeliness to the consideration of forwarding behavior. By dynamically analyzing the user interaction relationship network at moment t of the Weibo topic in the range of information dissemination, we mine the magnitude of the contribution of user v to user u’s blog interaction at moment t, which is expressed in terms of $\omega_{t}\left(u,v\right)$. The calculation formula is shown in Formula (9).

(9) $$\omega_{t}\left(u,v\right)=\frac{r_{t}\left(v\right)\times \lambda_{t}\left(u,v\right)}{out_{t}\left(v\right)}$$

where $r_{t}\left(v\right)$ is the amount of retweets directed to user u by user v at moment t, $out_{t}\left(v\right)$ is the number of retweets directed to other users under the topic by user v at moment t. The propagation ability of a user’s blog is a combination of user activity and interaction intensity. Users with strong influence dominate the dissemination of information, and the intensity of dissemination is higher than that of ordinary users. Blogs published by highly influential users are more likely to be retweeted and have a far greater influence than those published by ordinary users.

### 3.4. Relevance of User Interests

User interest is an important factor that influences the spread of information [36]. Different types of topics have different dissemination probabilities among user groups, and a certain type of topic can only hold users’ attention over time if it appeals to their interests. The correlation between a user’s interest and the topic represents whether the user will continue to follow the topic, engage in frequent interactions with it, and generate continuous influence. In the validity testing section of this paper, we verify the impact of the relevance between users and topics on users’ topic dissemination, showing that users with higher interest relevance engage in more frequent topic interactions and generate sustained influence compared to users with lower interest relevance. Some users engage in frequent topic discussions and interact with each other, and some even take on the role of powerful nodes, which can widen the path of diffusion and increase influence [37]. By analyzing the collection of blogs on Weibo under a certain topic during the collecting period, as well as the collection of previously posted blogs by users, we calculate the similarity of user interest and topics.

Generally speaking, a topic on Weibo is the collection of all blogs that address a single public opinion event [38]. Users typically post blogs on the same topic, and other users’ forwarding comments on these blogs are usually centered on the topic [39]. Based on this circumstance, we collect all blogs and the forwarding comments that correspond with them under a topic and put them together into a topic document set. The document set consists of a sizable number of brief documents that are all connected by a common topic.

Using this approach, we compile all user blogs published within the same time period into a user document set. In this study, we filter the obtained document set to remove noise and unnecessary information from the text to the greatest extent possible and then create a corpus of filtered texts by separating words and extracting related text feature words. Then we apply the TF-IDF algorithm to calculate the weight of the feature words and filter the final short text feature word ver. The formula for the TF-IDF algorithm is shown in Formula (10), where $f_{i,j}$ represents the frequency of occurrence of word j in document i, i.e., the ratio of the frequency of occurrence of word j in document i to the total number of words in document i. N is the total number of documents. $n_{j}$ represents the number of documents in which word j occurs. The formula indicates that when a word appears more frequently in one document and less frequently in other documents, the value of $tfidf_{i,j}$ is larger; that is, word j is more important for this document i. Therefore, this algorithm helps distinguish the importance of different words in a document [40].

(10) $$tfidf_{i,j}=\log(f_{i,j})\times \log\left(\frac{N}{n_{j}}\right)$$

Finally, we calculate the interest level of user u in the background topic based on the user feature embedding vector and the topic feature embedding vector. Cosine similarity is a method used to assess the degree of similarity between two vectors by their cosine values, which fits the characteristics of this paper to calculate the similarity between users and background topics. So, we use the cosine similarity to perform the calculation and obtain the similarity between user interest and topic features, which is expressed in terms of sim(u,topic). The calculation formula is presented in Formula (11).

(11) $$sim\left(u,topic\right)=\frac{ver\left(u\right)\cdot ver\left(topic\right)}{\left\|ver(u)\|\times \|ver(topic)\right\|}$$

where sim(u,topic) is the cosine similarity between the Weibo topic document set and the user document set. The greater the value of sim(u,topic), the higher the similarity between the user’s previous blog content and the Weibo topic, indicating that the user is more interested in such topics.

### 3.5. UWUSRank Model Construction

#### 3.5.1. Problem Analysis

This paper defines a dynamic model for calculating user influence based on the user’s own influence and interaction influence while also considering the similarity between user interests and the topic. To facilitate the explanation, Table 1 provides a list of symbols and their corresponding meanings in the dynamic user social network.

Through interactive behaviors, Weibo users connect with each other in the network, constructing a sophisticated information dissemination system based on social networks. In this paper, we investigate the extent of users’ effect on others in the social network by abstracting the Weibo social network into a dynamic network. Dynamic network $G=\left\{G_{1},G_{2},\ldots ,G_{T}\right\}$ refers to the network which consists of sets of graphs in time, among which $G_{T}=\langle U_{T},E_{T},W_{T},UA\rangle$ is the network topology at moment T, $U_{T}$ is the user set at the moment T, and $E_{T}$ and $W_{T}$ are the edge set and weight set at the moment, respectively. UA is the set of user attribute features, which includes information about the user’s followers, interaction information such as likes and retweets, and information about the user’s past blogs. Our task is to dynamically calculate the influence value of user u at different moments T.

#### 3.5.2. User Influence Definition Based on User’s Own Influence and Interactive Behavior Influence

In the process of Weibo opinion dissemination, the user’s ability to influence the intensity of information dissemination can be explored through their own influence, while the breadth of their ability to disseminate information can be explored through their interaction behavior. In this paper, we propose a reconstructed user influence evaluation algorithm, which improves upon the PageRank algorithm by combining the user’s own influence with their interaction behavior. This approach enables the calculation of the propagation influence $Affect_{t}\left(u\right)$ of user u in the topic evolution process. The calculation formula is shown in Formula (12).

(12) $$Affect_{t}\left(u\right)=\left(1-d\right)\times I_{0}\left(u\right)+d\times \underset{v\in O_{\left(u\right)}}{\sum }(Affect_{t}\left(v\right)\times \omega_{t}\left(u,v\right)\times \gamma_{t}\left(u,v\right))$$

where $Affect_{t}\left(u\right)$ is the propagation influence of user u at moment t, $I_{0}u$ is user u’s own influence, $Affect_{t}\left(v\right)\times \omega_{t}\left(u,v\right)\times \gamma_{t}\left(u,v\right)$ is the forwarding influence contributed by user v to user u at moment t, $O_{\left(u\right)}$ is the set of followers of user u, and d is the damping factor, which takes a value of 0.85.

The original PageRank algorithm assumes that each user is equally important and assigns the same initial influence score to all users. Moreover, each fan distributes its own score equally to the users it follows, which is not in line with reality. First of all, the initial influence of each user is different, and it should be determined by the user’s own influence. Secondly, although a user follows many users, it is impossible to treat all users equally, and the user will always be more interested in some of them and more willing to interact with their tweets, so the interaction contribution rate is different. Our core idea is that if a user follows another user with active interactions, then the user’s contribution to another user’s influence value should be higher than the default contribution of 1/m (if the user follows m users). Therefore, the algorithm proposed in this paper takes into account different users’ own information to determine their basic influence values, as well as the varying levels of contribution from different followers to the user’s influence. The stronger the interaction, the larger the corresponding followers will assign a larger proportion of the contribution value to the user. Meanwhile, this paper introduces user interest relevance, which takes into account the significant differences in the diffusion of different types of topic information among users and can more reasonably assess the actual influence of Weibo users.

#### 3.5.3. Model Construction with User Interest

A vast number of users’ communication behaviors constitute the general characteristics of information dissemination, and each user’s individual ability to disseminate information reflects their contribution to the process of disseminating Weibo topics [41]. User interest determines the user’s engagement and behavior continuity for a specific issue and is a key factor in the capacity to generate influence [42]. Therefore, the dissemination of different types of topic information among users differs significantly. We introduce the user’s interest in the participating topic as a potential variable, and we calculate the influence size $UWUSRank_{t}\left(u\right)$ of the topic participating user u at moment t using Formula (13).

(13) $$UWUSRank_{t}\left(u\right)=Affect_{t}\left(u\right)\cdot sim\left(u,topic\right)$$

#### 3.5.4. Algorithm Description

The UWUSRank algorithm dynamically characterizes user influence by considering both their own influence and interactive influence while also taking into account the similarity of their interests to the topic. The algorithm constructs a Weibo interaction network graph based on user data and calculates the influence of each user in the graph in turn. First, we calculate the user’s own influence as the initial value of influence. Then we calculate the interactive influence contributed by each follower in turn and compare the resulting influence value with the influence value of the previous round. If the difference is less than the threshold value, we exit the cycle and obtain the user’s influence value at this time; otherwise, we continue the calculation. Finally, we calculate the similarity between the user’s interest and the topic and multiply it by the influence value to obtain the final user influence value.

The main calculation process of the UWUSRank algorithm can be summarized as the following Algorithm 1.



**Algorithm 1: The UWUSRank Algorithm**
**Input**: Dynamic User Interaction Network $G_{T}$ **Output**: UWUSRank for each user **//Calculate the user’s own influence** **for all**  u
**in**
U **do** Compute act(u) using Equation (5) Compute res(u) using Equation (6) Compute aut(u) using Equation (7)
$I_{0}\left(u\right)=\phi_{1}\cdot act\left(u\right)+\phi_{2}\cdot res\left(u\right)+\phi_{3}\cdot aut\left(u\right)$ Initialize the influence value of each user as $I_{0}\left(u\right)$ **end for** **//Calculate user influence considering the user’s own influence and Interaction behavior** **for**
iter=1
**to**
max_iter
**do**       **while**
(u,v)∈ **E**       Compute $\omega_{t}\left(u,v\right)$ using Equation (9)       Compute $\gamma_{t}\left(u,v\right)$ using Equation (4)          $Affect_{t}\left(u\right)=\left(1-d\right)\times I_{0}\left(u\right)+d\times \sum_{v\in O_{\left(u\right)}}(Affect_{t}\left(v\right)\times \omega_{t}\left(u,v\right)\times \gamma_{t}\left(u,v\right))$           $\delta =\sum_{i=1}^{N}Affect_{iter}\left(u\right)-Affect_{iter-1}\left(u\right)$          **if** converged(δ) **then**              **break**          **end if**       **end while** **for all**
u in U **do**    **//Calculate the similarity between user interest and the topic content**                                             $sim\left(u,topic\right)=\frac{ver\left(u\right)\cdot ver\left(topic\right)}{ver(u)\times ver(topic)}$    **//Calculate the UWUSRank value**
                                    $UWUSRank_{t}\left(u\right)=Affect_{t}\left(u\right)\cdot sim\left(u,topic\right)$ **return**
$UWUSRank_{t}\;$ for each user


## 4. Experimental Evaluation

### 4.1. Data Preparation

Weibo is a social platform that contains numerous domains [32]. In order to verify the effectiveness and universality of the proposed method, we have selected five topics, including “Beijing Winter Olympic Games,” “The Itaewon Stampede Accident,” “Tangshan violence,” New Oriental bilingual online live,” FIFA World Cup Qatar 2022”. The experimental data covers multiple domains of public opinion, such as society, livelihood, judicial cases, entertainment, sports, and education. Taking the popular discussion time of general topics as 48 h per cycle, we use web crawlers to crawl the information of topic-related blogs for 18 cycles by searching the specified topic keywords. In addition, we collected interaction information such as followers, likes, forwards, and historical blogs from users during the specified period. The detailed data information crawled is as follows.

- User’s own information: User ID, Authentication, Followers set, Fans set, Published Blog ID, and Release time;
- Blog information: Author ID, Blog ID, Blog content, Number of likes, Number of comments, Number of forwards, and Release time;
- Passive interactive information of users: User ID, Blog ID, Forwarder ID, and Forwarded time;
- Active interaction information of users: User ID, Blog ID which is forwarded, and Forward time.

The number of blogs published by users can be obtained through the collection of Blog IDs published by users. Adding the number of likes and reposts in blog information can determine the value of act(u). By obtaining the number of comments, likes, and forwards in a user’s blogs, the value of res(u) can be obtained. The aut(u) can be obtained from the authentication status in the user’s own information. At this point, the influence value $I_{0}\left(u\right)$ based on the user’s personal attributes is obtained. In time period t, through the user’s passive interaction information, we can get in-degree neighbors and the number of forwarded blogs. Similarly, through the user’s active interaction information, we can get out-degree neighbors and the number of forwards. Then the value of $\omega_{t}\left(u,v\right)$ and $\gamma_{t}\left(u,v\right)$ can be obtained, thus obtaining the value of $Affect_{t}\left(u\right)$. This process iterates continuously until convergence. By analyzing the blog content in the context of the topic and the blog content posted by the user, the user’s interest relevance value in the context of the topic can be obtained. Finally, the value of $UWUSRank_{t}\left(u\right)$ can be obtained.

Through the above crawling process, we finally obtained a social network dataset of 271,451 posts and 167,334 users. The number of blogs and users per topic is shown in Table 2.

**Figure 4:**
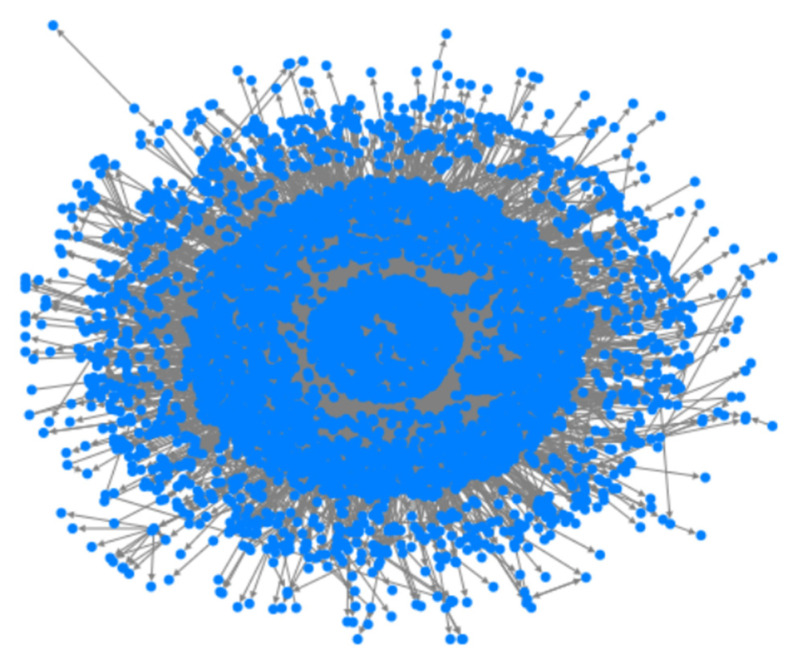
CCTV News’s Weibo forwarding relationship network.

In the following, we provide an example analysis of the interaction relationship among users using the blogs about “The Itaewon Stampede Accident” published by CCTV News. The result is shown in Figure 4, where each node represents a Weibo user, and interactions form connected edges between nodes. The more connected edges the node has to the outreach, the more frequent the interactions are, and the more important the node is. These interactions can serve as a reflection of user impact. Compared with ordinary users, strongly influential users’ blogs are often reposted by a large number of other users in the network. The original blog is located at the center and spreads outward in layers. During the data collection period, including CCTV News, a total of 7872 users participated in the dissemination of the blog. First of all, from the graph, it can be seen that users with high influence are distributed near the central user, and the number of reposts at various communication levels is decreasing, indicating that users with high levels of closeness are more likely to pay attention to their posts and participate in their dissemination. Secondly, the faster users engage in topic interaction, the greater the number of reposts they receive compared to those who engage in subsequent interactions. This indicates that engaging in interactive behavior in a short period of time after a blog post can generate stronger interaction heat and higher influence. Therefore, the interaction timeliness and connection tightness proposed in this study can accurately represent the weight of interaction behavior between users in social networks.

### 4.2. Experimental Protocol

In order to validate the effectiveness and accuracy of the UWUSRank in measuring user influence, we selected TwitterRank, FansRank, and PageRank for comparison. The reasons for selecting these algorithms are as follows: The TwitterRank algorithm considers the type of topic content and network structure as well but lacks consideration of user interaction behavior; FansRank focuses on the participating users in the process of information dissemination, which is also a common way of opinion guidance; PageRank is a classic web ranking algorithm, whose core idea is often applied in the process of modern information dissemination model research.

The three main types of experimental protocols in this paper are as follows:

1. Comparison of the rationality of user influence ranking: This experiment analyzes the reasonableness of user influence ranking by cross-validating the results of influence ranking obtained by different calculation methods;
2. Comparison of user influence coverage: This experiment evaluates the performance of different models qualitatively by comparing the change curve of user node group coverage area under Top-k variation;
3. Validity Analysis: This experiment examines the effect of user interest, time factor and each indicator of the user’s own influence on the user influence value in the context of the topic. It includes the following sub-protocols:
  - The influence of user’s interest relevance on the results in the context of the topic: Compare the influence of interested users and non-interested users on blog dissemination impact;
  - The influence of time factor on user influence: Analyze the change of users’ influence on the timeline by comparing the difference in users’ influence in different time periods;
  - The influence of users’ own indicators on user influence: By calculating the user activity, blog response and user authentication of the top 10 users in the standard set, we analyze whether the three indicators in the user’s own influence can be used as evaluation criteria for user influence calculation.

### 4.3. Analysis of Experimental Results

#### 4.3.1. Rationalization of User Influence Ranking

This study conducts a quantitative analysis and assessment of the user rankings generated by each algorithm using three indicators: precision, recall, and F-value, in order to test the rationality of user influence ranking. The actual user impact ranking is determined by cross-validating the results of these four algorithms, and the calculation formula is shown in Formula (14).

(14) $$R_{2}=\left(R_{UWUSRank}\cap R_{TwitterRank}\right)\cup \left(R_{UWUSRank}\cap R_{FansRank}\right)\cup \;\left(R_{UWUSRank}\cap R_{PageRank}\right)\cup \left(R_{TwitterRank}\cap R_{FansRank}\right)\cup \;\left(R_{TwitterRank}\cap R_{PageRank}\right)\cup \left(R_{FansRank}\cap R_{PageRank}\right)$$

where $R_{UWUSRank}$ is the set of user influence ranking under the UWUSRank algorithm, $R_{TwitterRank}$ is the set of user influence ranking under the TwitterRank algorithm, $R_{FansRank}$ is the set of user influence ranking under the FansRank algorithm, $R_{PageRank}$ is the set of user influence ranking under the PageRank algorithm, and $R_{2}$ is the final set of user influence rankings.

Precision refers to the proportion of relevant instances among all retrieved instances. In the context of user influence mining, precision can be defined as the proportion of correctly identified influential users among all users identified as influential by the algorithm [43]. Taking the UWUSRank as an example, the precision rate of user influence mining under Weibo topics can be defined as shown in Formula (15).

(15) $$Precision_{UWUSRank}=\frac{\left|R_{UWUSRank}\cap R_{2}\right|}{R_{UWUSRank}}$$

Recall measures the completeness of the results measured by the algorithm [44]. Taking the UWUSRank as an example, the recall rate of user influence mining under Weibo topics is defined as shown in Formula (16).

(16) $$Recall_{UWUSRank}=\frac{\left|R_{UWUSRank}\cap R_{2}\right|}{R_{2}}$$

F-value is a comprehensive evaluation index that balances the impact of Precision and Recall. The definition of the F-value is shown in Formula (17).

(17) $$F_{UWUSRank}=\frac{2\times Precision_{UWUSRank}\times Recall_{UWUSRank}}{Precision_{UWUSRank}+Recall_{UWUSRank}}$$

**Figure 5:**
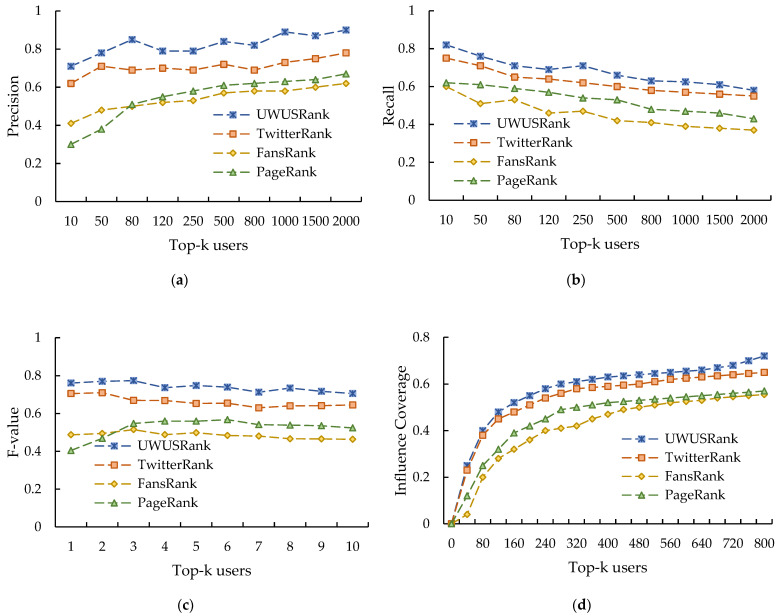
(**a**) Precision comparison results; (**b**) Recall comparison results; (**c**) F-value comparison results; (**d**) Impact coverage comparison results.

By empirically evaluating the average performance of these four methods under topics, Figure 5a–d demonstrates the experimental findings of the average precision, recall, and F-value under various Top-k, respectively. The results show that:

- According to the findings of the precision comparison shown in Figure 5a, the overall precision of each algorithm increases as the data volume of the user rating dataset increases. Among them, the precision of UWUSRank outperforms TwitterRank, PageRank and FansRank under different Top-k values. TwitterRank algorithm takes second place. The FansRank algorithm has a higher accuracy than the PageRank algorithm at Top 10, but as the number of users increases, the accuracy of FansRank is the worst;
- From the comparison results of the recall rate in Figure 5b, we can see that UWUSRank also outperforms TwitterRank, PageRank and FansRank, and the effect is better;
- From the comparison results of comprehensive indexes in Figure 5c, we can see that the UWUSRank can obtain the best ranking results of user influence, followed by the TwitterRank and PageRank, while FansRank performs the worst.

The above results demonstrate the strong correlation between users’ influence and their own influence and capacity for interaction. UWUSRank performs better when measuring the influence of Weibo users and mining highly influential users. In addition, solely using the number of user followers as an evaluation indicator of user influence has poor practical results.

#### 4.3.2. User Influence Coverage

Strong influencers in social networks are able to reach more users [45]. According to this feature, this paper selects the user influence coverage as an evaluation index. Influence coverage refers to the coverage of other nodes that are cumulatively affected by information diffusion after a node in a social network publishes information. The calculation formula is shown in Formula (18).

(18) $$CR\left(k\right)=\frac{\sum_{k=1}^{N}CN\left(k\right)}{N}$$

where CR(k) indicates the influence coverage of the top k user nodes. CN(k) is the number of other nodes influenced by the user node ranked k, and N is the set of all user nodes in the topic network. After evaluating the influence coverage of the top 800 users under each algorithm during the blog interaction, the experimental results are shown in Figure 5d. According to the influence coverage evaluation findings, the user influence coverage trends across various algorithms are observed to be essentially the same. However, UWUSRank significantly outperforms the remaining three algorithms, and the top 800 users mined achieve the best influence coverage. Conversely, the influence coverage of the FansRank is the lowest, indicating that although some information dissemination networks have a significant number of originating nodes with fans, there is little fan activity overall, limited fan contact, and insignificant influence from the nodes.

#### 4.3.3. Validity Test

1. Analysis of the influence of user interest on blog dissemination

**Figure 6:**
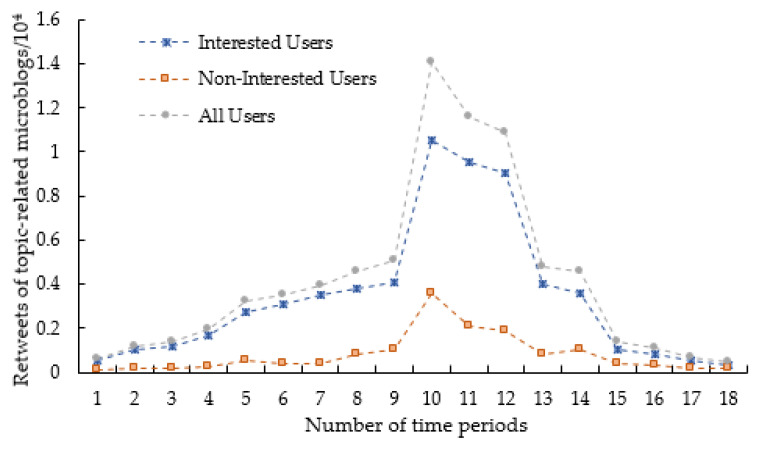
Statistics of retweets by two types of users under Weibo topics.

In order to study the impact of users’ interests on their communication behavior, this paper classifies users by calculating the similarity between the content of their blogs and the content of the topics. According to the research findings of Yang et al. [46], users with a similarity value greater than 0.8 are referred to as interested users, while those with a similarity value lower than 0.8 are referred to as uninterested users. As an example, we analyze the change in the number of tweets related to the “The Itaewon Stampede Accident” topic retweeted by interested and uninterested users over a period of 48 h, as shown in Figure 6.

The points in Figure 6 represent the retweets of the two types of users for the blogs under the topic in each time period. Observing Figure 6, we can see that the increase in discussions on the Weibo topic page influences the retweets of both types of users during the topic dissemination cycle. Among them, the growth of retweets of interested users is significantly higher than that of non-interested users. Meanwhile, compared with non-interested users, the trend of interested users’ retweets is significantly similar to the trend of overall discussion volume retweets under topic pages. This shows that interested users are more engaged in the topic, which encourages the faster diffusion of a topic and steadily strengthens the influence of online opinion dissemination. It verifies that adding user interest relevance helps to discover users who can exert influence in the process of Weibo opinion dissemination;

2. Analysis of the influence of time factor on user influence

**Figure 7:**
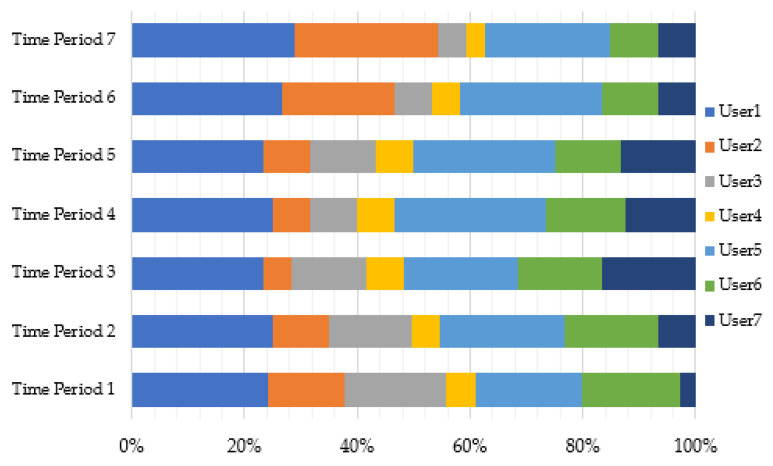
Change of user influence in different time periods.

The UWUSRank can track how user influences change over time. Here we take “The Itaewon Stampede Accident” topic as the data set, set a time period to 12 h, and have a total of seven time periods for the statistical cycle. Then we use the UWUSRank algorithm to calculate the user influence values under each of the seven time periods. Throughout the statistical cycle, a total of 10,049 users participated in the topic discussions, from which we extracted the top seven users in the overall ranking. These users have consistently maintained a sizable influence and fan base throughout the study period. Figure 7 displays the changes in the influence values of the top seven users in different time periods.

The influence values of users vary with time, and highly influential individuals are not always more influential than other users. Figure 7 demonstrates that user 1 and user 5 have a significant and enduring influence. The reason is that the two users, as the initiators of many hot topics, have built up a high-stickiness user network, and the public hot event blogs published by them are more likely to trigger widespread discussion. User 2 had a relatively high influence ranking in the first two time periods but then dropped significantly due to the lack of blogs in the middle period. The opposite is true for User 7, indicating that the activity of the central users is a prerequisite for strong interaction. The influence of User 3 and User 6 has been steadily increasing because they keep updating their blogs, and the content of the updated blogs is related to recent hot topics. This kind of regular participation in trending issues might attract more network members and increase awareness. It demonstrates that user influence is dynamic. As a result, adding the time factor to user analysis is more in accordance with social network features, which can increase the precision of influence computation.

3. Analysis of the influence of each user’s own attributes on user influence

This section also uses “The Itaewon Stampede Accident” topic as the data set, with a time period of 48 h. We have calculated the user activity, blog response and user authentication of the top 10 users in the influence ranking in the standard set and have also calculated the user’s own influence value proposed in this paper. As shown in Table 3, it is found that most of the users with high influence are certified Weibo users, and most of them are officially certified users, which shows that the credibility of users can largely reflect the influence of users. At the same time, users with high user activity and blog responsiveness have a higher reach. Therefore, user activity, blog responsiveness and user trustworthiness can accurately describe the user’s own influence and can be the criteria for user influence evaluation.

## 5. Conclusions

User influence evaluation is a crucial aspect of social network research, and it plays an active role in estimating social opinion and guiding social hotspots. In this paper, we explore the dissemination mode of Weibo public opinion in the new media era, analyzing both users’ own influence and interactive influence. At the same time, we introduce users’ interest preferences and propose a dynamic calculation model of user influence based on interest preferences by improving the PageRank algorithm based on the information dissemination process of users’ dynamic interaction mechanism.

Our experiment results on a large-scale Weibo dataset demonstrate that the proposed method can effectively improve the accuracy of user influence calculation in Weibo communities and uncover highly influential user groups. In the future, we plan to use the public opinion information dissemination network as the basis of our research, from which we can explore the group sentiment characteristics in the process of event dissemination and find out the pattern of sentiment contagion. Furthermore, we will study the role of textual emotion in the process of change of Weibo users’ influence to effectively improve the level of public opinion guidance.

## Figures and Tables

**Table 1:** Symbol Description.

Notations	Definition
$G_{T}=\langle U_{T},E_{T},W_{T},UA\rangle$	Dynamic user social network
U	All users who participated in the topic
E	Collection of interactive edges in the network
W	Connection-side forwarding weights
UA	User attribute feature set
$\gamma_{t}\left(u,v\right)$	Tightness of the connection between user u and v at time t
$\lambda_{t}\left(u,v\right)$	Timeliness of user ’s interaction with v
act(u)	User Activity at time t
res(u)	Blog Response
aut(u)	User Trustworthiness
$I_{0}\left(u\right)$	Influence value based on the user’s own influence
$\omega_{t}\left(u,v\right)$	Interaction contribution rate at time
$Affect_{t}\left(u\right)$	Influence value based on the user’s own quality and interaction ability at time t
sim(u,topic)	Similarity of user u’s interest in the topic
$UWUSRank_{t}\left(u\right)$	User influence values calculated by UWUSRank

**Table 2:** Experimental data set.

Topic	Number of Blogs	Number of Users
The Itaewon Stampede Accident	64,098	39,944
Tangshan violence	55,900	33,540
New Oriental bilingual online live	31,297	17,526
FIFA World Cup Qatar 2022	57,392	36,156
Beijing Winter Olympic Games	62,764	40,168
Total	271,451	167,334

**Table 3:** Top 10 users of standard set ranking.

Ranking	User ID	User Activeness	Blog Response	Authentication Status	Self-Quality Impact Value
1	7516679696	16,467.73	0.64976929	1	5489.79298
2	5463794433	9479.19	0.56641124	1	3160.25
3	3266943013	7031.94	0.45384404	1	2344.46378
4	1686546714	4665.00	0.51390066	1	1555.50463
5	2602644737	3807.33	0.25319541	0.5	1269.36218
6	2656274875	2876.15	0.30239771	1	959.15
7	2482557597	1484.98	0.10767825	0.5	495.20
8	1644114654	1104.08	0.07664361	1	368.39
9	6059492811	806.44	0.03749561	0.25	268.91
10	6191604322	670.85	0.03866419	0.25	223.71

## Data Availability

The data presented in this study are available upon request.
